# Definition of the effector landscape across 13 phytoplasma proteomes with LEAPH and EffectorComb

**DOI:** 10.1093/nargab/lqae087

**Published:** 2024-07-30

**Authors:** Giulia Calia, Alessandro Cestaro, Hannes Schuler, Katrin Janik, Claudio Donati, Mirko Moser, Silvia Bottini

**Affiliations:** Faculty of Agricultural, Environmental and Food Sciences, Free University of Bolzano, 39100 Bolzano, Italy; Research and Innovation Centre, Fondazione Edmund Mach, 38010 San Michele all’Adige, Italy; INRAE, Institut Sophia Agrobiotech, Université Côte d’Azur, CNRS, 06903 Sophia-Antipolis, France; Research and Innovation Centre, Fondazione Edmund Mach, 38010 San Michele all’Adige, Italy; Institute of Biomembranes, Bioenergetics and Molecular Biotechnologies (IBIOM), National Research Council (CNR), 70126 Bari, Italy; Faculty of Agricultural, Environmental and Food Sciences, Free University of Bolzano, 39100 Bolzano, Italy; Competence Centre for Plant Health, Free University of Bolzano, 39100 Bolzano, Italy; Institute for Plant Health, Molecular Biology and Microbiology, Laimburg Research Centre, 47141 Pfatten-Vadena, Italy; Research and Innovation Centre, Fondazione Edmund Mach, 38010 San Michele all’Adige, Italy; Research and Innovation Centre, Fondazione Edmund Mach, 38010 San Michele all’Adige, Italy; INRAE, Institut Sophia Agrobiotech, Université Côte d’Azur, CNRS, 06903 Sophia-Antipolis, France

## Abstract

*‘Candidatus* Phytoplasma’ genus, a group of fastidious phloem-restricted bacteria, can infect a wide variety of both ornamental and agro-economically important plants. Phytoplasmas secrete effector proteins responsible for the symptoms associated with the disease. Identifying and characterizing these proteins is of prime importance for expanding our knowledge of the molecular bases of the disease. We faced the challenge of identifying phytoplasma's effectors by developing LEAPH, a machine learning ensemble predictor composed of four models. LEAPH was trained on 479 proteins from 53 phytoplasma species, described by 30 features. LEAPH achieved 97.49% accuracy, 95.26% precision and 98.37% recall, ensuring a low false-positive rate and outperforming available state-of-the-art methods. The application of LEAPH to 13 phytoplasma proteomes yields a comprehensive landscape of 2089 putative pathogenicity proteins. We identified three classes according to different secretion models: ‘classical’, ‘classical-like’ and ‘non-classical’. Importantly, LEAPH identified 15 out of 17 known experimentally validated effectors belonging to the three classes. Furthermore, to help the selection of novel candidates for biological validation, we applied the Self-Organizing Maps algorithm and developed a Shiny app called EffectorComb. LEAPH and the EffectorComb app can be used to boost the characterization of putative effectors at both computational and experimental levels, and can be employed in other phytopathological models.

## Introduction

Plants live in a constantly changing environment and have developed high phenotypic plasticity including responses and adaptations to environmental factors (1,2). In the case of pathogen infection, interactions are based on a molecular dialog between the pathogen and its host. Plants and pathogens are engaged in an arms race where plants have evolved stratagems to detect the bio-aggressor while the latter aims to bypass the host's immune system (3–6). Plants modulate their immune response, cell signaling, metabolism and even development upon pathogen attack by acting on regulation of its transcriptome (7–9). This modulation of gene expression relies on the secretion of specific pathogenicity factors called effector proteins. The effectors' arsenal and their delivery system vary from species to species, although they have the common aim of interfering with the host’s metabolism to their advantage, and to hamper the immune system, enhancing pathogen survival and, as a consequence, the development of the disease (10–14).

The increasing availability of genome sequences of plant pathogens has allowed dramatic advances in the characterization of pathogenicity mechanisms and the development of tools to improve effector prediction. For instance, EffectorO is a machine learning model exclusively trained on the N-terminus of oomycete effector proteins (15). Another example is EffectorP1.0 to 3.0, an ensemble of machine learning models to predict both effectors and their localization (apoplastic or cytoplasmic) trained on both fungi and oomycetes to search for characteristic enrichment of amino acids and their properties (16–18). Deepredeff is a convolutional neural network trained on sequences of bacteria (Gram positive and Gram negative), fungi and oomycetes (19). DeepT3 combines different deep-learning algorithms to predict effectors secreted by the bacterial type III secretion systems (T3SSs) in Gram-negative bacteria (20). Finally, EffectiveT3 is a machine learning model to predict whether effector proteins are secreted by the T3SS (21). Despite these tools allowing advances in knowledge of effectors for some plant pathogens, there is still a lack of prediction methods for several pathogens, hampering a global characterization of pathogenic mechanisms.

A clear example of this category is the ‘*Candidatus* Phytoplasma’ genus, a group of plant-pathogenic, phloem-restricted, bacteria assigned to the Mollicutes class, cell wall-less and pleomorphic bacteria, ranging from 0.2 to 0.8 μm in size (22–25). Phytoplasmas are associated with diseases in a large number of crops, ornamental plants and trees that result in altered development and huge yield losses in plants (26–29). Symptoms of the disease include, among the most common, witches' broom agglomerates of young branches, increased proliferation of shoots, yellowing of the leaves, virescence (flower organs become green), phyllody (flowers develop into leaf-like flowers), dwarfism, reduction in size and tasteless fruits (24,30,31). The transmission from plant to plant occurs by insects, which become vectors of the pathogen once they acquire the phytoplasma during phloem feeding (32–34). The pathogen behavior of phytoplasmas and the difficult challenge of cultivating them under *in vitro* conditions have hindered experimental studies focusing on the identification of effector proteins. Similarly, because of their high amino acid sequence variability, the *in silico* identification of effector proteins exclusively based on the overall sequence similarities is inefficient (14). As a consequence, until a few years ago, only certain types of effector proteins had been identified in phytoplasmas (35). The main feature of those candidate effectors was the presence of signal peptides, restricting the diversity of effectors to only a few classes. The possible role in disease development of the following classes of effectors was mainly studied by expressing them in transgenic plants. TENGU, a small secreted peptide encoded by onion yellow phytoplasma, causes dwarfism and altered flower structures (36). SAP05 which interferes with plant vegetative growth is a secreted protein found from ‘*Ca*. P. asteris’ (37,38). SAP11-like proteins, first identified from aster yellow witches’ broom phytoplasma, cause abnormal proliferation of young shoots and changes in leaf shape (37,39). SAP54 and PHYL1 are two homologous effectors that belong to the phyllogen family and cause flower malformations including phyllody, virescence and proliferation (37,40,41).

Nowadays, standard methods for effector identification in phytoplasmas are only based on the presence of a signal peptide, fairly reconstructing the phytoplasmas’ secretome. However, it is important to consider that not all the secreted proteins are effectors and that some effector proteins are secreted by non-classical pathways (42,43). Moreover, it is shown that in Gram-positive bacteria, ancestors of phytoplasmas, the hydrophobic region of signal peptides is longer than usual, making them more similar to transmembrane regions and undetectable by software specifically designed for signal peptide prediction (44). Thus, recent methods rely on the combined predictions by software designed for both signal peptide and transmembrane domain detection (44,45), leading to a large number of candidates to be tested without any prioritization in assessment. Despite the recent attempt of Carreón-Anguiano *et al.* (46) to build up an ad hoc pipeline suitable for phytoplasma effectors, there is still an urgent need for a tailored and reliable method to characterize, predict and prioritize phytoplasma effectors.

To address these needs, we developed LEAPH (ensemb**L**e model for **E**ffector cl**A**ssification in **PH**ytoplasmas) a computational method composed of an ensemble of four supervised learning models to capture distinct sets of features and efficiently predict putative effector proteins in phytoplasmas. Indeed, LEAPH predicts effectors with ∼97% accuracy, outperforming existing prediction methodologies tailored for both phytoplasmas and other pathogens. The ensemble model was trained on 479 proteins coming from > 50 ‘*Ca*. Phytoplasma’ species and then applied, as a use-case scenario, on 13 proteomes (ranging from 327 to 730 proteins), allowing us to identify a comprehensive landscape of putative candidate effectors. We used the Self-Organizing Map (SOM) algorithm to describe the properties of this landscape, which combines clustering and dimensionality reduction to embed the protein sequence similarities based on the feature profiles. Therefore, neighboring points on the map represent proteins sharing similar features, providing the first reference map for phytoplasma effectors. We developed a user-friendly Shiny application, called EffectorComb, to investigate the effector protein map. Overall, LEAPH and EffectorComb offer the possibility to predict, interpret and explore the resulting protein candidates, thus boosting the experimental validation process from the very beginning.

## Materials and methods

### Training datasets

For sequence selection and curation of the training dataset, we used the UniProt database (release 2022_01) (47), selecting proteins uniquely belonging to the phytoplasmas TAXID 33926.

#### Positive dataset

**Figure 1.**
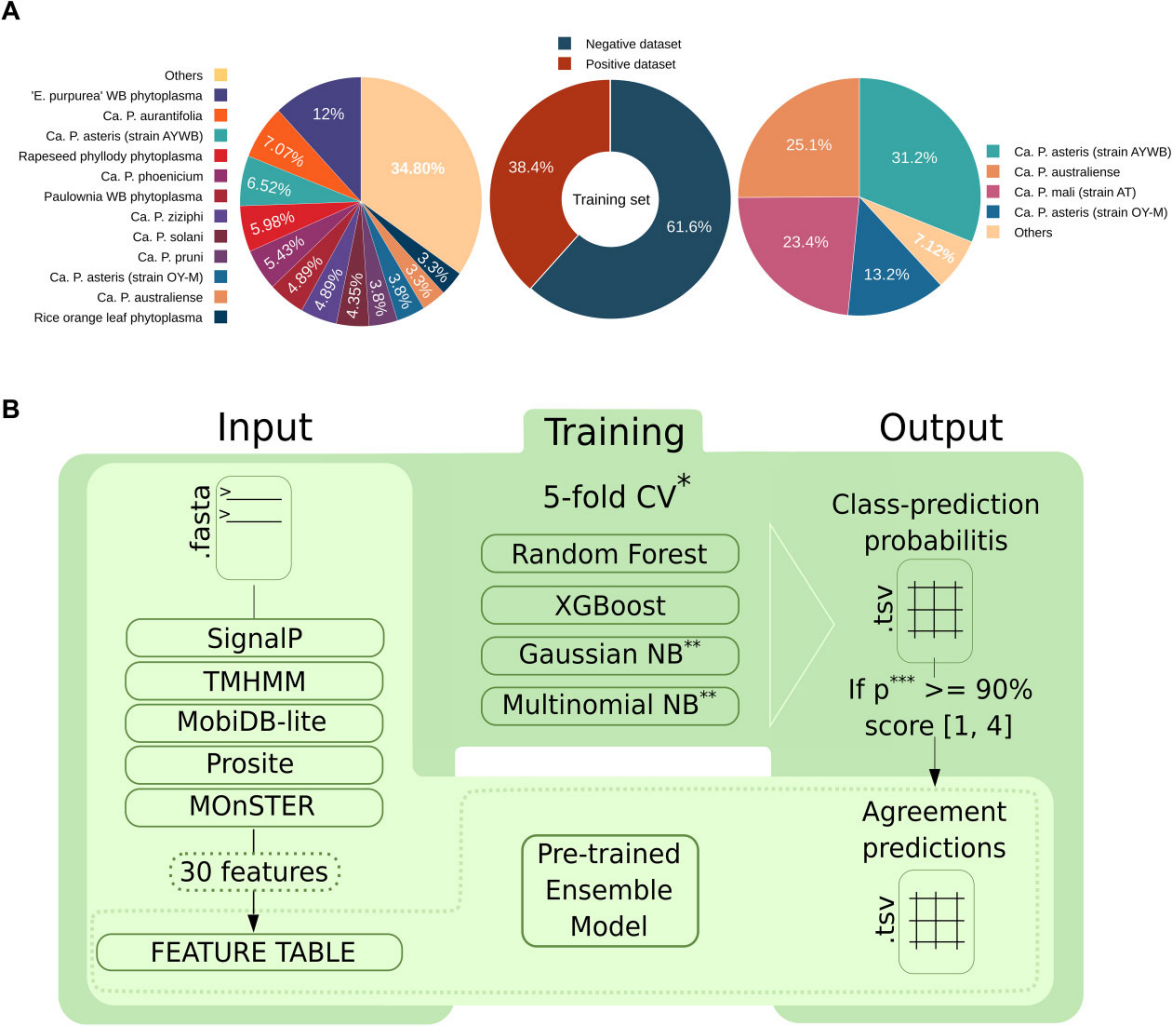
Training dataset composition and LEAPH workflow. (**A**) Training dataset composition of the positive and negative set proportion (in the center) and phytoplasma species for the positive dataset (on the left) and negative dataset (on the right). Refer to Supplementary Tables S1.1 and S1.2 for details. (**B**) LEAPH workflow. The dark green box highlights the training process while the light green box represents the application process of LEAPH; the dashed box depicts the particular use case in which a feature table is already available, thus ready to be used as input for the pre-trained LEAPH model.

The positive dataset is created using two filters. Firstly the field ‘Protein Names’ is searched using the AND operator and each of the following regular expressions: ‘*Effector*’, ‘*TENGU*’, ‘*SAP54*’, ‘*SAP11*’, ‘*SAP05*’ and ‘*PHYL1*’. Secondly, the resulting list is pruned from records that have the terms ‘putative’ or ‘fragment’ in the ‘Protein names’ field. The result is a set of 174 proteins from 53 phytoplasma species (Figure 1A). Another 10 experimentally validated proteins are retrieved from Hoshi *et al.* (36) for TENGU; Bai *et al.* (37) for SAP54; Bai *et al.*, 2008 and Kube *et al.* (37,48) for SAP11; Bai *et al.* (37) for SAP05; and Maejima *et al.* (41) for PHYL1. In total, the positive dataset accounts for 184 protein sequences (Supplementary Table S1.1).

#### Negative dataset

The negative dataset is constructed by manual inspection of UniProt database entries having evidence for revision in the ‘Protein existence’ field AND having a function not related to the known effectors’ activity (e.g. no ‘*SVM*’ or ‘*trigger factor*’ in ‘Protein Name’ field). The remaining list is checked for the absence of a cross-reference with the PHI-base database (49,50), meaning that there is no known interaction between these proteins and those of the host. The negative dataset is finally composed of 295 non-effector proteins from 12 phytoplasma species (Supplementary Table S1.2; Figure 1A).

### Proteome datasets

We used LEAPH to predict putative effector proteins in 13 phytoplasma proteomes: ‘*Ca*. P. asteris’ (strain AYWB-UP000001934 and OY-UP000002523), ‘*Ca*. P. australiense’ (UP000008323), ‘*Ca*. P. mali’ (strain AT-UP000002020), ‘*Ca*. P. oryzae’ (strain Mbita1-UP000070069 and S10-UP00024934), ‘*Ca*. P. phoenicium’ (strain ChiP-UP00023867 and SA213-UP00003708), ‘*Ca*. P. pruni’ (strain CX-UP00003738), ‘*Ca*. P. tritici’ [2568526557 from JGI-GOLD (51)], ‘flavescence dorée’ [CP097583.1 from NCBI-GeneBank (52)], ‘*Ca*. P. ziziphi’ (CP091835.1 from NCBI-GeneBank) and ‘*Ca*. P. asteris’ M3 (CP015149.1 from NCBI-GeneBank). The proteomes are retrieved from UniProt if not indicated otherwise. The main characteristics of the 13 proteomes are summarized in Supplementary Table S2.

### LEAPH method description

The LEAPH workflow consists of four main steps described hereafter and shown in Figure 1B. Briefly, in step 1, LEAPH takes as input the amino acid sequences of interest in FASTA format and computes the 30 features, described in the following paragraph, yielding as output the feature table. During the training process (step 2), the feature table is used as input for four classifier models to be trained. After a 5-fold cross-validation process, each of the best models is used to finally assign a class probability to every protein in the overall training dataset (step 3). LEAPH predicts a protein to be putatively pathogenic if at least one of the four models gives a class probability > 90% and thereby assigns a score ranging from 1 to 4 to each protein based on the models' agreement (step 4). The pre-trained model can be used to predict putative pathogenicity proteins in any set of protein sequences, producing an output table that contains the resulting classification along with model agreement, mean prediction probability and the corresponding amino acid sequence for each protein.

#### Feature calculation

The first step of LEAPH is the annotation of protein sequences and the extraction of features to describe protein sequence properties. We included a total of 30 features: (i) sequence length; (ii) signal peptide presence [using the D-score of SignalP 4.1 software (53) configured as in the work of Garcion *et al.* (44)]; and (iii) transmembrane (TM) domain presence [TMHMM2.0 (54)]. Specifically, we included different aspects of the prediction of TM regions, namely the number of predicted TMs, (iv) the expected number of amino acids in TMs, (v) the number of expected amino acids in the first 60 positions of TM helices, (vi) the probability that the N-terminus of the protein is in the cytoplasm and (vii) a warning for possible misprediction of the TM regions [when feature (vi) is > 10], representing a potential signal peptide region [mispredicted transmembrane region (mTMR)]. Since effector proteins can be composed of intrinsically disordered regions (IDRs) (55), we also predicted (viii) the eventual presence and the length of IDRs [MobiDB-Lite1.0 (56,57)]. Finally, we added 22 features concerning putative characteristic motifs of effectors: (ix–xx) by using Prosite1.86 (58); we calculated the occurrences of functional protein motifs belonging to the positive dataset, thus each of the 22 features is the sum of the respective predicted motif in a protein sequence; (xxi–xxx) by using MOnSTER (59), we obtained cluster of Prosite motifs (CLUMPs) considering their physicochemical characteristics. From MOnSTER application, we extracted six features representing the occurrence of each selected CLUMP and four additional features, namely the occurrence of CLUMPs in four consecutive bins of 25% of the protein sequence. The configuration parameters for all tools used to obtain the 30 features and the main descriptions are indicated in Supplementary Table S3.

#### Models

LEAPH is based on an ensemble learning approach that uses four classification algorithms: random forest (60) [RandomForestClassifier() from sklearn.ensemble], XGBoost (61) [XGBClassifier() from the xgboost python library], Gaussian naive Bayes (62) [GaussianNB() from sklearn.naive_bayes] and multinomial naive Bayes (63) [MultinomialNB() from sklearn.naive_bayes]. The version of scikit-learn library is 1.1.1 for all the methods (64). See the following section and Supplementary Table S4 for more information on usage and parameter settings.

#### Cross-validation and hyperparameter tuning

Because of the size and the unbalanced distribution of proteins between classes in the training set, we applied the stratified 5-fold cross-validation method (StratifiedKFold from scikit-learn 1.1.1). Each model is trained and tested five times on a different subset of the training data respecting class proportion (80% of data for the training set and the remaining 20% for non-overlapping test sets).

We performed a hyperparameter tuning via GridSearchCV from scikit-learn 1.1.1, for each fold, and for each classifier except for Gaussian naive Bayes which was trained with the default parameters. With the grid search, the parameters are tested from a specified set of values. The starting parameters used in the training process, which differ from the default ones, are included in Supplementary Table S4. Altogether cross-validation and hyperparameter tuning allow selection of the best model for each classifier algorithm to be used to predict new putative pathogenicity proteins.

To support the reproducibility of the machine learning method of this study, the machine learning summary table (Supplementary Table S4) is included in the supporting information as per DOME recommendations (65).

#### Classification method

The best model for each classifier obtained from the previous phase is used to finally assign a class probability to every protein in the overall training dataset: if for at least one model the class probability is ≥ 90%, then the protein is considered as putatively pathogenic. LEAPH assigns a score ranging from 1 to 4 to each protein based on the models’ agreement. The pre-trained model can be used to predict novel putative pathogenicity proteins in any set of proteins, yielding an output table containing the classification prediction, the model agreement, the mean prediction probability and the corresponding amino acid sequence for each protein.

### Performance measures

To assess the performance of the prediction models used in this study, we used the following metrics: accuracy, precision, recall and F_1_ score defined as:


(1) \begin{eqnarray*}Accuracy = \frac{{TP + TN}}{{TP + TN + FP + FN}}\end{eqnarray*}

(2) \begin{eqnarray*}Precision = \frac{{TP}}{{TP + FP}}\end{eqnarray*}

(3) \begin{eqnarray*}Recall = \frac{{TP}}{{TP + FN}}\end{eqnarray*}

(4) \begin{eqnarray*}{{F}_1} = \frac{{2\left( {Precision{\mathrm{*}}Recall} \right)}}{{\left( {Precision + Recall} \right)}}\end{eqnarray*}

Where TP, TN, FP and FN refer to true positives, true negatives, false positives and false negatives, respectively. These measures are calculated using the sklearn.metrics library.

### Benchmark of effector prediction tools

The state-of-the-art method commonly used in prediction of phytoplasma effectors consists of a combination of two predictors: SignalP4.1 and TMHMM2.0. Specifically, a protein is predicted as putatively pathogenic if both a signal peptide and an mTMR are predicted. This method is included in the benchmark as the reference method for phytoplasma effectors. Recently, Carreón-Anguiano *et al.* published the first pipeline that identifies effectors in phytoplasmas, PhyEffector (46). This pipeline adds to the previous two tools, also Phobius (66), BLASTP (67) and SecretomeP2 (68), thus we included it in the benchmarking. We also compared the performance of LEAPH with other effector prediction tools on the training set. We considered EffectorP3.0 (18), which is mainly suitable for fungi and oomycete putative pathogenicity proteins, EffectorO (15), tested for oomycete putative effector prediction, and Deepredeff (19), used for putative pathogenicity protein prediction in bacteria, fungi and oomycetes. All the tools are used with default parameters or, where possible, in a configuration suitable for bacteria.

### Feature importance calculation

We assessed the feature importance for each classification algorithm using the SHapely Additive exPlanations (SHAP) approach (69). SHAP is widely used for explainable machine learning and gives a more comparable spectrum of feature importance across different models. It measures the contribution of each feature to the final output using game theory concepts and feature permutations, assigning a SHAP value to each feature. Compared with the classical feature importance measurements, this additive feature method relies on maintaining the accuracy of the model, dealing with missing features and consistency in model changing for the same data.

### SecretomeP2.0

To predict non-classically secreted proteins, we used SecretomeP2.0 (68). Since the Gram-positive bacteria model is currently unavailable for the latest version of the tool, we used the Gram-negative bacteria model. We split the sequences into 100 sequences per fasta file [using seqkit split (70) and setting the parameter ‘-s 100″] to use the web server, and we cross-checked each prediction with the presence of a signal peptide predicted by both SignalP4.1 and TMHMM2.0, as per SecretomeP2.0 web service recommendations.

### Exploratory and functional analysis

We performed a principal component analysis (PCA) on scaled values (MinMaxScaler from scikit-learn 1.1.1) of the 30 protein features described in the section ‘Feature calculation**’**, and InterProScan5 (71) to predict protein domains associated with a known biological function on the predicted putative pathogenicity proteins identified by LEAPH. We then explored the differences in protein domains that occur in at least 1% of the sequences, according to the secretion modality of the predicted proteins.

### Effector protein map

We used SOM (72) to exploit the properties of the putative pathogenicity proteins identified by LEAPH from 13 phytoplasma proteomes. To build up the SOM, we firstly scaled the values of each feature (MinMaxScaler from scikit-learn 1.1.1), excluding mTMR as a categorical, thus not suitable, feature and then used the aweSOM R package, which outputs a dynamic map. Through SOM visualization, points on the map in the same hexagonal cell share a similar feature vector and thus are indeed similar to each other. To find the optimal number of hexagonal cells to create the map, we tried different sizes of lattice grids from 8 × 8 up to 11 × 11, where we stopped because the 10 × 10 achieved the best balance between the errors (Quantization, Topographic and Kaski-Lagus) minimization and variance explained (see Supplementary Table S5 for further details).

### EffectorComb app

We developed an interactive Shiny app to investigate the obtained SOM. The app allows the user to retrieve a set of pathogenicity proteins predicted by LEAPH with properties of interest along with their sequence identifier and amino acid composition. It is also possible to download SOM interactive image .html files. EffectorComb can be also used to project new proteins, predicted by LEAPH application, on the pre-obtained SOM for a deep result exploration and comparison.

### Implementation

LEAPH is implemented in python3.8.10 language (available at https://www.python.org/). The build-up process is done using jupyter-notebook v6.4.8 (73) while the final LEAPH model and running software is a python3.8.10 script that can be used as stand-alone software or by executing a singularity v3.7 container (74) which includes all the required dependencies. EffectorComb was first implemented as R 4.3.2 (available at https://www.r-project.org/) script and then embedded into a Shiny app (v1.8.0, available at https://shiny.posit.co/, R version) and provided within a singularity v3.7 container. Usage instructions and scripts are freely available at https://github.com/Plant-Net/LEAPH-EffectorComb.git.

## Results and discussions

### A comprehensive collection of phytoplasma effectors and features to describe their sequence characteristics

To fulfill the need for a method tailored for predictions of phytoplasma effectors, we developed LEAPH, a machine learning ensemble model. Careful selection of the training set and features to use for classification purposes are crucial in machine learning applications. Therefore, by performing extensive literature and database mining, we collected 184 protein sequences from 53 ‘*Ca*. Phytoplasma’ species that make up the ‘positive dataset’. The ‘negative dataset’ is composed of 295 proteins whose function is not related to the activity of the known effector and/or no interactions with host plant proteins are reported (Figure 1A; Supplementary Tables S1.1 and S1.2). Intending to build a classifier for the prediction of novel effectors, we calculated 30 features to represent the salient characteristics of their sequences (Supplementary Table S3). We included eight features that describe the protein sequence properties and the mode of secretion, 12 features summarizing the presence of protein domains important for plant invasion and infection and 10 features relating to the presence of characteristic sequence motifs found by using MOnSTER (59). To inspect whether these features are discriminant of putative effector properties, we plotted the distribution of each, for the positive and negative datasets, respectively (Supplementary Figure S1). Twenty-four features show a significantly different distribution (Mann–Whitney test, *P*-value < 0.05) by comparing the two datasets. However, we decided to include all features because non-significantly different features can be specific to a small class of putative effectors, too small to reach statistical significance when including all the effector proteins together.

Overall, both the datasets and the features are suitable for setting up a classifier based on machine learning models.

### The four classifiers included in LEAPH captured distinct features to predict pathogenicity proteins

Because of the wide variety of hosts and infection symptoms, we expect phytoplasmas’ effectors to have different characteristics, thus the hypothesis is that different learning models would be able to capture diverse properties, yielding a more comprehensive prediction. Therefore, we used an ensemble learning composed of two tree-based algorithms, namely random forest and XGBoost (60,61), and two naive Bayes classifiers, i.e. a Gaussian and a multinomial model (62,63). We fed the four classifiers with our training dataset and measured their performances on the test set (Supplementary Table S4). Overall, the four models showed very good and comparable performance both within the cross-validation folds and between methods, ranging from 95% to 99% for the four measures used: precision, recall, accuracy and F_1_ score (Supplementary Table S6; Supplementary Figure S2). The best performing model was selected for each classifier method and included in the ensemble approach that we call LEAPH (ensemb**L**e model for **E**ffector cl**A**ssification in **PH**ytoplasmas). To each candidate pathogenicity protein predicted with LEAPH, a score ranging from one to four is associated, corresponding to the number of classifiers that agree on the outcome.

**Figure 2.**
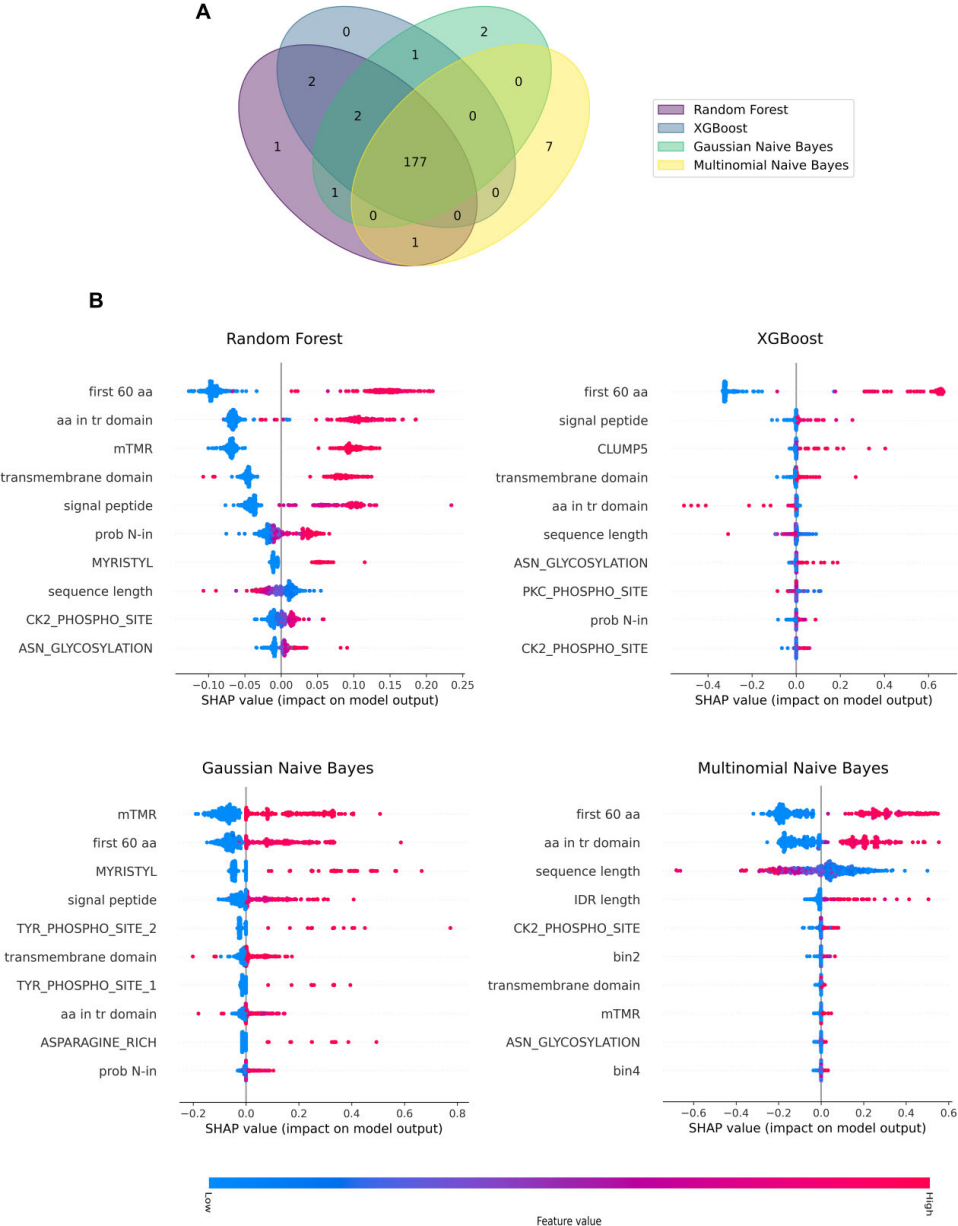
LEAPH and SHAP results on the training dataset. (**A**) Venn diagram representing the agreement of prediction on the training set from the four models included in LEAPH, namely random forest model (purple ellipse), XGBoost (dark blue ellipse), Gaussian naive Bayes model (aquamarine ellipse) and multinomial naive Bayes model (yellow ellipse). (**B**) The first 10 features in terms of SHAP value importance for each model included in LEAPH. Refer to Supplementary Figure S3 for the complete representation of SHAP importance.

In Figure 2A, we report the Venn diagram showing the agreement of the prediction obtained by the four models. Most candidate effectors were predicted by the four models (96%), as expected. Interestingly, the two naive Bayes classifiers, and mainly the multinomial model, were able to identify putative effectors that were not identified by the two tree-based models, supporting our hypothesis that different classifiers might capture different patterns of candidate pathogenicity proteins. To further investigate this direction, we calculated the feature importance for each classifier by using the SHAP algorithm (69) which is a game theory approach to explain the output of any machine learning model. Indeed, as shown in Figure 2B and Supplementary Figure S3, we observe a quite different spectrum of features, with the highest contribution scores depending on the classifier. While the feature describing the first 60 amino acids of the protein sequence is found among the top two for all methods, the other features seem to contribute differently, depending on the classifier. In particular, we observed that only for the multinomial naive Bayes, the sequence length has a high impact on the model output. On the other hand, for the Gaussian naive Bayes model, the mTMRs make the greatest contribution. Moreover, the top 10 rank of the contributing features for all the models include the presence of three functional motifs related to protein modification sites: myristoylation, glycosylation and phosphorylation sites. This means that the occurrence of these motifs in the protein is a discriminative predictive sign for each of the included models.

**Figure 3.**
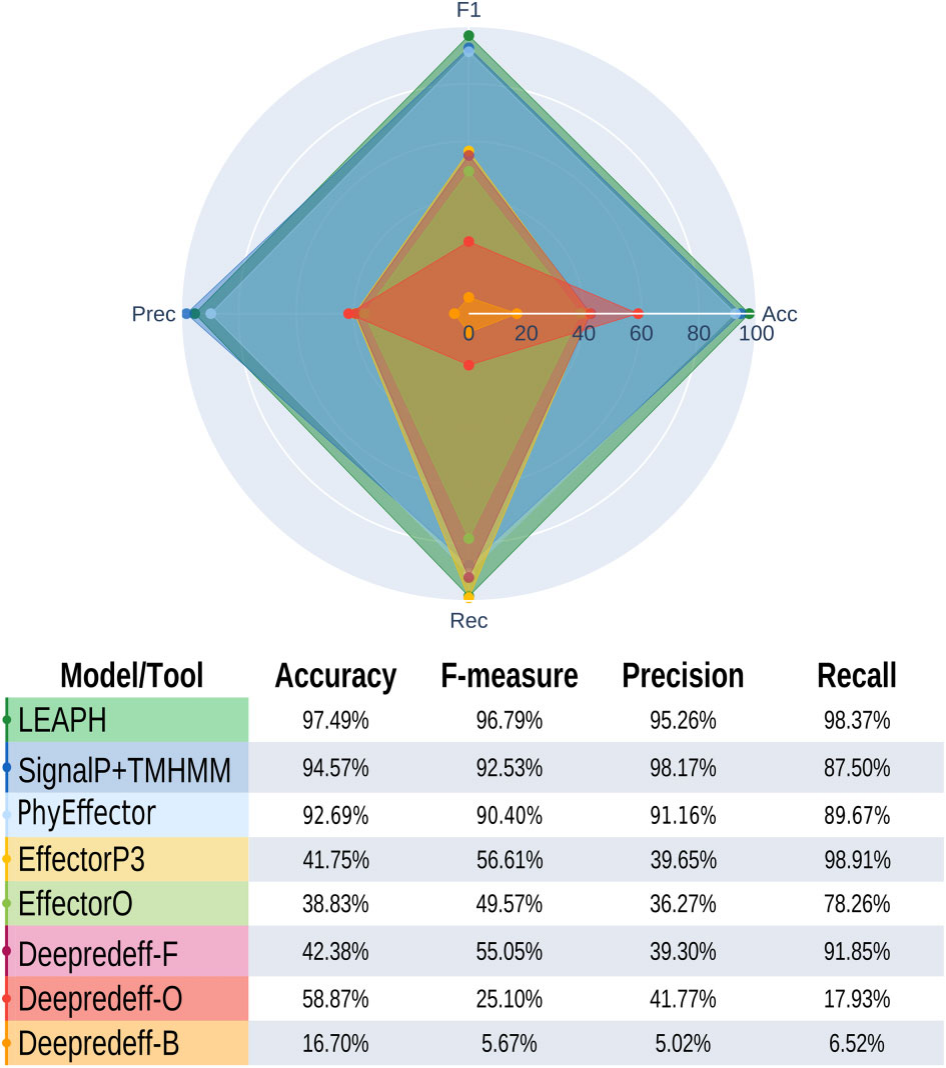
Performances of LEAPH and state-of-the-art tools for effector prediction. Performances of each tested state-of-the-art tool compared with LEAPH performance. For each tool, the percentage of accuracy, F-measure, precision and recall after the application on the training set is reported.

Altogether these results show that the four classifiers included in LEAPH can identify potential pathogenicity proteins with different characteristics and confer good performance on the test dataset.

### LEAPH outperformed other methods with regard to the prediction of effectors in phytoplasmas

To assess the performances of LEAPH compared with state-of-the-art tools for effector prediction, we compared LEAPH application to the training dataset with five classifiers available for effector prediction, namely SignalP4.1 (53) in combination, or not, with TMHMM2.0 (54) which we consider here the conventional method to predict putative effectors in phytoplasmas; the newly implemented PhyEffector (46) tailored for phytoplasma effector prediction; EffectorO (15); EffectorP3.0 (18); and the three models for Deepredeff (19) that are built to predict putative effectors in other species (Supplementary Table S7; Figure 3). LEAPH outperformed all the other methods by considering the four metrics used in this study, achieving a recall of 98.4%, thus predicting only three false negatives. In this context, the minimization of false negatives is crucial, to minimize the neglecting of putative true effectors. Importantly, the tool LEAPH also achieved good precision (95.3%), thus implying a low number of false positives as well (nine in total). Although experimental validation is central to validate putative candidate effectors predicted by computational methods, the low number of both false negatives and false positives increases the confidence in the prediction and will permit a more successful experimental validation.

Application of EffectorP3.0, EffectorO and Deepredeff-fungi showed quite good recall but very low values for the other metrics. This is due to the high number of false positives identified by those methods that lower the accuracy (ranging from 38.8% to 42.4%) and the precision (between 36.3% and 39.6%). On the other hand, the use of EffectorP3.0 and Deepredeff-fungi do not identify many false negatives, with a recall of 98.9% and 91.8%, respectively. Lower performances are obtained for EffectorO with a recall of 78.3%. The tools Deepredeff-oomycetes and Deepredeff-bacteria showed very poor performance.

The three combinations of SignalP4.1 with TMHMM2.0, namely SignalP4.1, SignalP4.1 + TMHMM2.0 and SignalP4.1/TMHMM2.0 (in Supplementary Table S7) applied to our data achieved comparable performances mainly in terms of accuracy concerning LEAPH. However, their recalls are lower than the value achieved by our tool, suggesting that these methods might miss potential bona fide effector candidates. Since SignalP4.1 and TMHMM2.0 by definition can identify only putative classically secreted effectors, we hypothesize that these false negatives can be potential non-classically secreted pathogenicity proteins that are captured by LEAPH thanks to the four classifiers. As expected, PhyEffector application showed good performances on the training set, reaching 92.7% accuracy, 91.2% precision, 89.7% recall and an overall F_1_ score of 90.4% (Supplementary Table S7; Figure 3). Surprisingly, accuracy and F_1_ score were found to be slightly lower than those achieved by SignalP4.1 + TMHMM2.0.

Altogether these results highlight the importance of developing a more flexible and informative method tailored for phytoplasmas to identify bona fide candidate effectors and rule out EffectorP3.0, EffectorO, Deepredeff and PhyEffector from further comparisons.

### LEAPH predicts classical, classical-like and non-classical secreted putative pathogenicity proteins from 13 phytoplasma proteomes

**Figure 4.**
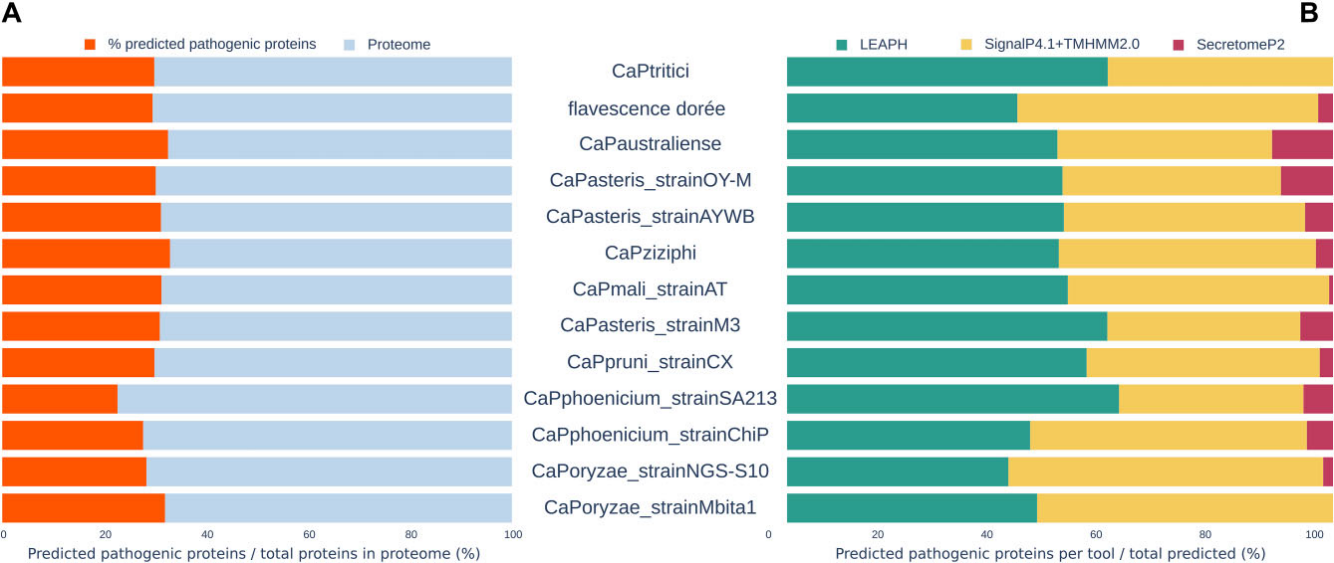
Putative pathogenicity proteins predicted by LEAPH from 13 phytoplasma proteomes. (**A**) Percentage of putative pathogenicity proteins predicted by LEAPH (orange bars) compared with the total percentage of proteins (gray bars) for the corresponding phytoplasma proteome. (**B**) Comparison of the proportion of proteins predicted only by LEAH (green bars), predicted by the SignalP + TMHMM method and LEAPH (yellow bars) or by SectetomeP2.0 and LEAPH (magenta bars).

To test the capability of LEAPH to identify potential novel effectors, we selected 13 phytoplasma proteomes from 10 ‘*Ca*. Phytoplasma’ species with different characteristics in terms of the number of proteins, 16S group, type of symptoms and number of hosts (Supplementary Table S8; Figure 4). On average, LEAPH predicts as putative pathogenicity proteins ∼30 ± 2.7% of the proteomes except for three strains: CaPhoenicium_SA213, CaPhoenicium_ChiP and CaPoryzae_NGS-S10, for which we have found 22.63, 27.65 and 28.32%, respectively (Figure 4A).

To characterize the properties of these putative pathogenicity proteins, we compared them with the prediction of SignalP4.1 + TMHMM2.0 and SecretomeP2.0 (68). SignalP4.1 + TMHMM2.0 is used to identify proteins having a signal peptide, usually present in classically secreted proteins, whereas SecretomeP2.0 predicts non-classically secreted proteins. Importantly, both methods predict whether the protein is secreted or not without information on its pathogenicity. LEAPH predicts between 40% and 61% of putative effector proteins that were not identified by the other two methods (Figure 4B). Between 33% and 58% of LEAPH putative effectors also show the signal peptide and the mTMR identified by TMHMM2.0, thus implying that they are classically secreted. These results pinpoint that LEAPH, despite the fact that it was trained on a dataset composed mainly of classically secreted effector proteins, can capture characteristics to identify potential effectors beyond the classically secreted ones. On the other hand, very few effectors predicted by LEAPH were also predicted by SecretomeP2.0. This was expected because the model used to run SecretomeP2.0 was suitable for Gram-negative bacteria, but no model was available for Gram-positive bacteria. According to Gao *et al.* (75), SecretomeP2.0 underestimates non-classically secreted proteins when using the Gram-negative bacteria model for Gram-positive bacteria. Interestingly, between 77% and 89% of putative effectors predicted exclusively by LEAPH contain only the mTMR (without the signal peptide), supporting the hypothesis that phytoplasma effectors might have a peculiar signal peptide located in the so-called sequence variable mosaic (SVM) regions of some proteins (76).

Finally, we checked whether the known validated phytoplasma effectors were correctly classified by LEAPH. Eleven classically secreted effectors were identified and validated by previous studies: two SAP11-like proteins in two different ‘*Ca*. Phytoplasma’ species [‘*Ca*. *P*. asteris’ strain AYWB and ‘*Ca*. P. mali’ strain AT (37,48)], SAP54 [‘*Ca*. P asteris’ strain AYWB (37)], SAP05 [‘*Ca*. P. asteris’ strain AYWB (37)], PHYL1 [‘*Ca*. *P*. asteris’ strain OY-M (41)], TENGU [‘*Ca*. P. asteris’ strain OY-M (36)], SWP1, SWP11, SWP12, SWP16 and SWP21 [‘*Ca*. P. tritici’ (77–80)]; and six non-classically secreted effectors in the strain ‘*Ca*. P. ziziphi’: ncSecP3, ncSecP12, ncSecP14, ncSecP22, ncSecP9 and ncSecP16 (75) (Supplementary Table S9). Remarkably, LEAPH could not only correctly identify the 11 classically secreted effectors, but also four out of six non-classically secreted validated effectors (ncSecP3, ncSecP12, ncSecP14 and ncSecP22). This result strengthens the potential of LEAPH to identify putative effectors independently of their type of secretion.

### Description of the effector protein landscape predicted by LEAPH

**Figure 5.**
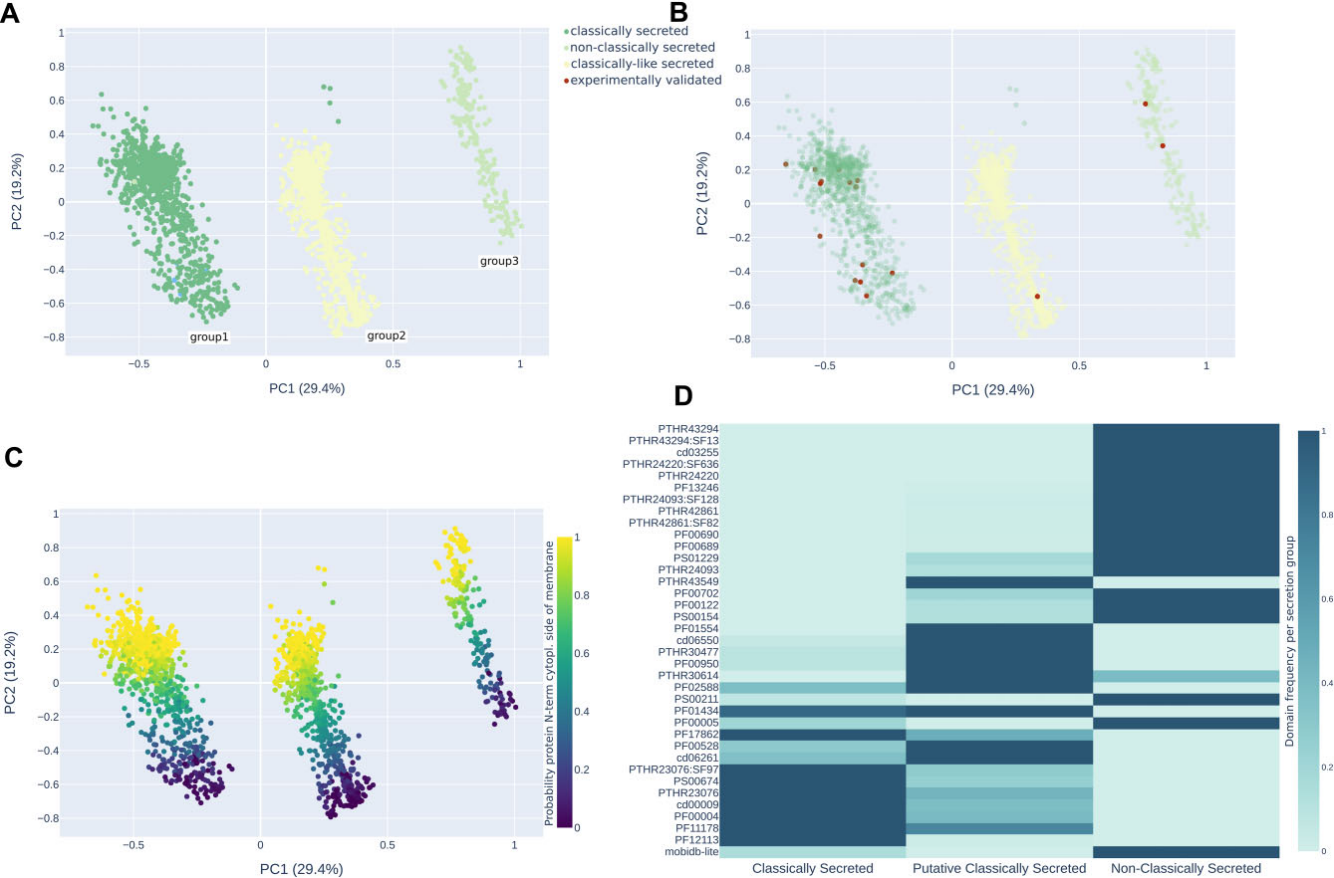
Description of the effector protein landscape predicted by LEAPH. (**A**) PCA of predicted pathogenicity proteins by LEAPH from 13 phytoplasma proteomes colored by secretion methodology; group1 (dark green points) predicted by SignalP4.1 to bear a signal peptide (classically secreted), group2 (light yellow) contains an mTMR in the first 60 amino acids of the sequence from TMHMM2.0 and no prediction of signal peptide by SignalP4.1 (classically like secreted) and group3 (light green points) do not have the signal peptide and the mTMR (non-classically secreted). (**B**) The same as (A) with the position of the 15 experimentally validated effectors from the literature (dark red dots). (**C**) Same as (A) colored by the probability that the N-terminal region of the protein is in the inner part of the membrane. (**D**) The abundance of each protein domain predicted by InterProScan in each PCA group.

To further characterize the putative effectors predicted using LEAPH, we performed a PCA on protein features (see the Materials and methods). In Figure 5, we observe three distinct groups. To understand whether these groups could represent some property of protein sequences, we colored them according to the type of secretion. We defined as ‘classically’ secreted, those proteins in which both a signal peptide and mTMR are predicted; ‘classically like’ secreted, those having only the prediction for the mTMR; and ‘non-classically’ secreted, those proteins in which neither signal peptide nor mTMRs are predicted (Figure 5A). The three groups identified with the PCA respectively follow the three secretion modalities. Thus, we checked where the validated effectors are located in the putative pathogenicity protein landscape identified with the PCA (Figure 5B). The 11 classically secreted validated effectors (two SAP11 in two different strains, SAP54, SAP05, PHYL1, TENGU, SWP1, SWP11, SWP12, SWP16 and SWP21) were localized in the cluster of classically secreted as expected. Surprisingly, among the four non-classically secreted validated effectors, only two were found in the corresponding cluster, namely ncSecP3 and ncSecP12. Unexpectedly, by inspecting the protein sequences, we found that ncSecP14 contains the signal peptide and the mTMR, and ncSecP22 only the mTMR, thus confirming their localization on the PCA.

Then, we mapped other properties on the three PCA groups. Interestingly, inspecting the second principal component, we noticed that the putative pathogenicity proteins found using LEAPH are stratified by the probability for the N-terminus to have a cytoplasmic location, as shown in Figure 5C. When coloring by species, we observed that there are no specific clusters, as expected, meaning that pathogenicity protein properties are not species specific (Supplementary Figure S4A). In Supplementary Figure S4B, we can observe that proteins are stratified by sequence length. Surprisingly we observe that few proteins from the classically secreted and non-classically secreted groups have a size larger than expected. Thus, we mapped these proteins on the sequence length distribution of the respective proteomes to see whether these sizes could be potential outliers or not (Supplementary Figure S4C). We observed that very few putative effectors, mainly predicted as classically secreted, map on the tails of these distributions.

To investigate other possible peculiar properties of LEAPH-predicted putative pathogenicity proteins, we used InterProScan5 (71) to predict protein domains in the three classes of secretion. We considered only domains with an occurrence of at least 1% for at least one of the secretion classes, for a total of 37 protein domains. As shown in Figure 5D, classically secreted putative effectors mainly possess domains characteristic of both SVM proteins, AAA+ ATPases and FtsH proteins. SVM proteins were reported for the presence of a modified secretion signal (76). Recently, it has been demonstrated that the AAA+ and Ftsh domains have a role in pathogenicity for ‘*Ca*. P. mali’ (81,82). Classical-like secreted candidate pathogenicity proteins, on the other hand, are mostly characterized by periplasmic-binding proteins or ABC transporters and only slightly enriched in AAA+ and Ftsh domains. Although ABC transporters are found ubiquitously in eukaryotes and prokaryotes, they play a crucial role in pathogenesis and virulence in pathogen bacteria (83). There are different types of ABC transporters [10 according to Zeng and Charkowski (84)] and this might be linked to the presence of different ABC-related domains characterizing the non-classically secreted group of predicted pathogenicity proteins. In this class, ABC transporters are less frequent concerning the P-ATPases and especially the IDRs. P-ATPases, particularly copper exporter P-type ATPases, are reported to play a major role in the virulence of diverse pathogenic bacteria even if the underlying mechanisms remain partially understood (85–89). These findings suggest that putative effectors are distinguished not only by the secretion mode, but also by other characteristics related to virulence properties embedded in the sequence composition.

### SOM clustering allowed us to build a pathogenicity protein reference map for phytoplasmas

**Figure 6.**
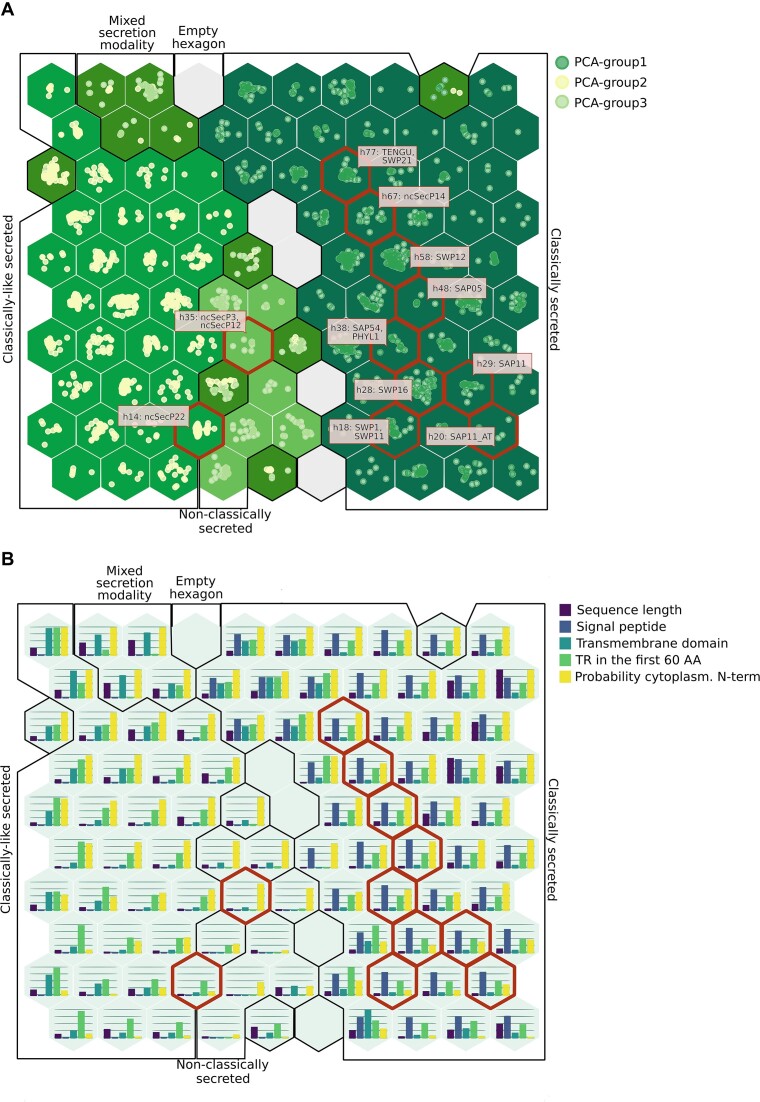
Pathogenicity protein reference map for phytoplasmas. (**A**) LEAPH predicted proteins projected on an SOM and colored by their secretion group: classically secreted (dark green points and dark green hexagons), classically like secreted (light yellow points and green hexagons), non-classically secreted (light green points and light green hexagons) and mixed secretion modality (olive green hexagons). Dark red contours indicate the hexagons containing the validated effectors from the literature. Empty gray hexagons represent profiles of features that are not represented by the predicted proteins. (**B**) The same as (A) showing the abundance of features for each hexagon.

To study whether other properties are characteristic of subclasses of pathogenicity proteins beyond the type of secretion, we performed a clustering analysis on the LEAPH-predicted proteins using the SOM model (72). This analysis allowed us to create a 2D map composed of a 10 × 10 lattice where each hexagonal cell is characterized by a particular combination of features and closer cells have more similar properties than cells further away. Thus, the 2093 putative pathogenicity proteins, including the 15 validated effectors, identified with LEAPH across 13 phytoplasma proteomes are distributed into the map and associated with a specific hexagonal cell according to their sequence properties. Consistent with PCA, the two main properties that stratify the hexagons in the lattice are the signal peptide and the mTMR on the *x*-axis, and the probability for the N-terminus to have a cytoplasmic location on the *y*-axis (Figure 6A). Therefore, we can visualize on the map the hexagons that correspond to the three groups found by the PCA: 49 hexagons for classically secreted, 31 for classically like and seven for non-classically secreted putative pathogenicity predicted proteins. Furthermore, eight hexagons that contain proteins from both groups of classically like and non-classically secreted and five empty hexagons can be identified on the map.

Interestingly, the 15 validated effectors are projected in nine different and close hexagons [including ncSecP14 proposed by Gao *et al.* (75) as non-classically secreted but having the signal peptide as discussed in previous paragraphs] (Figure 6A). As expected the two homologous phyllogen proteins (PHYL1 and SAP54) are in the same hexagon (h38). Similarly, SWP21 and TENGU are in the same hexagon (h77). The SAP11-like proteins do not fall in the same hexagon but in hexagons next to each other, suggesting more variability in the sequence properties of proteins in this class depending on the phytoplasma species. Intriguingly, SWP1 and SWP11, which have different roles in disease development (77), are in the same hexagon (h18). Interestingly, both interact with the TEOSINTE BRANCHED 1/CYCLOIDEA/ PROLIFERATING CELL FACTOR 1 and 2 (TCP) transcription factors, thus suggesting shared sequence properties to enhance the interaction with similar partners to investigate further (90). The two non-classically secreted validated effectors (ncSecP12 and ncSecP3) are mapped in the same hexagon (h35), also suggesting very similar sequence properties. Importantly, LEAPH predicted new putative effectors which are mapped in the same hexagons as the biologically validated effectors offering novel potential effector candidates with similar sequence characteristics to be experimentally validated.

### EffectorComb: a Shiny app to inspect the map of the pathogenicity proteins

To further inspect other properties and characteristics of the sequences contained in the pathogenicity protein map, we have developed a Shiny application, called EffectorComb, enabling us to show which features are enriched in each hexagon (Supplementary Figure S5). For instance, we notice that the enrichment of phosphorylation sites is mainly present in hexagons collecting togethger classically secreted putative pathogenicity proteins. Specifically, hexagon h70 is characterized by a high presence of the domain cAMP- and cGMP-dependent protein kinase phosphorylation site. Similarly, the Protein kinase C phosphorylation site and Casein kinase II phosphorylation site were found predominantly in h90. Tyrosine kinase phosphorylation sites 1 and 2 were also found enriched in h25, h99, h100 and h48. Protein phosphorylation is a widely used mechanism in bacteria to adapt to changes in their environment (where the conditions can alter rapidly), but it is also used for intercellular communication. The *N*-glycosylation site, specific to the consensus sequence Asn–Xaa–Ser/Thr, was found enriched in both classically like (h74) and classically secreted (h89) candidate pathogenicity proteins. Bacteria have evolved diverse glycosylation systems for pathogenesis. Bacterial glycosylation not only allows adhesion to the host cell but also functions to modulate crucial host cellular processes (91). Notably, glycosylation has been reported as an important process in the interaction between the ‘flavescence dorée’ phytoplasma and its insect vector (92), but also of phytoplasma strains and host plants (93,94). Interestingly the hexagon h74 is also characterized by the presence of the *N*-myristoylation site. *N*-myristoylation is an irreversible protein lipidation; in plants it is recognized as a major modification and concerns nearly 2% of all plant proteins (95,96). Attachment of a myristoyl group increases specific protein–protein interactions leading to the subcellular localization of myristoylated proteins with their signaling partners (97). Intriguingly, Medina-Puche *et al.* (98) identified novel mechanisms involving protein relocation due to the presence of the *N*-myristoylation site from the plasma membrane to chloroplasts that are utilized in plants for defense responses against pathogens. Furthermore, amidation sites were found mainly in the hexagons h10 and h40. C-terminal amidation reduces the overall charge of a peptide; therefore, its overall solubility might decrease. Modifications at the C-terminus have already been shown to have consequences on the pathogenicity of phytoplasmas; for instance, deletions at the C-terminus of the effector SWP1 impaired the induction of witches’ broom (99). The enrichment of this domain might suggest that changes in protein solubility are important for phytoplasma effectors and might reflect distinct functions also depending on the different hosts (insect/plant). The leucine zipper domain is found only in the classically secreted putative pathogenicity proteins lying in h19. This pattern is present in many gene regulatory proteins, making this cluster very interesting for further investigation (100). Finally, the ‘RGD’ tripeptide that has been shown to play a role in cell adhesion (101) is abundant in both classically (h82 and h98) and non-classically (h1, h17 and h25) predicted pathogenicity proteins.

Overall, the SOM map allowed us to identify distinct classes of putative pathogenicity proteins with particular properties probably linked to their function and type of interaction with the plant and/or insect.

## Conclusions

The few known biologically validated effectors in phytoplasmas are implicated in a huge spectrum of different functions, ranging from the regulation of plant morphogenesis to attracting insect vectors. They possess a variety of strategies to manipulate the host plants. Therefore, it is unlikely for phytoplasma effectors to be described by a few common characteristics. However, the most widely used method to select pathogenicity candidates for phytoplasmas is to screen protein sequences and look only for the presence of the signal peptide. Recently, a novel tool was introduced, PhyEffector, consisting of a pipeline of four different tools aimed at the identification of signal peptides and TM domains, and inspecting sequence similarity (46). Despite the promising performances of this tool, the results still lack a wider comprehension of effector physicochemical characteristics, a system by which to prioritize and evaluate the accuracy of the predictions and tools to help speed up the experimental validation process. Leveraging four machine learning classifiers coupled with a novel voting score for assessing the prediction score, we have developed LEAPH specifically conceived for phytoplasma effector candidate prediction. We have shown that using LEAPH, it is possible to predict putative effector candidates with high reliability and outperform existing tools not adapted for phytoplasmas. A major advantage of LEAPH consists of its ability to predict candidate pathogenicity proteins independently by the presence of the signal peptide and with many different sequence characteristics. Furthermore, as our knowledge of effector proteins increases, the early stage of LEAPH can be refined by constituting a novel training dataset to re-train the models to improve their performances.

The application of LEAPH on 13 phytoplasma proteomes led to the identification of 2093 putative candidates showing sequence characteristics linked to virulence and pathogenicity. To investigate the properties of those proteins, we used the SOM algorithm and obtained the first pathogenicity protein map for this pathogen. To provide an easy exploration of the pathogenicity protein map, we have developed an easy-to-use Shiny application called EffectorComb. To the best of our knowledge, this is the first time that a comprehensive characterization of putative pathogenicity proteins has been provided for phytoplasmas. The use of this map goes beyond the identification of groups of putative pathogenicity proteins having similar properties, but can be used as a reference map to project new predicted pathogenicity proteins from other proteomes in a predictive perspective. Therefore, when using this map as a reference to analyze a new sample, the positioning of each predicted pathogenicity protein of a new proteome on the 2D plane can be used to assess its pathogenicity properties and overall sequence characteristics. The pathogenicity protein map can also be used to accelerate the choice of novel candidates for biological validation, for instance by focusing on proteins in the same group of known validated effectors or on groups showing particular sequence properties of interest.

Overall, an ensemble of four learning model never used before in phytoplasma effector prediction, the introduction of a prediction prioritization score based on model agreement, the delineation of the phytoplasma effector reference map coming from a wide range of phytoplasma proteomes and the possibility to easily explore it, interact with it and retrieve protein sequences from it, make LEAPH and EffectorComb novel and valuable tools to improve our understanding of effectors in plant–phytoplasma interactions. Finding novel experimentally validated effectors can be used to set up novel methods to improve plant resistance to these devastating bacteria.

## Supplementary Material

lqae087_Supplemental_Files

## Data Availability

The datasets supporting the conclusions of this article are included within the article and its additional files. Project name: LEAPH-EffectorComb Project home page: https://github.com/Plant-Net/LEAPH-EffectorComb.git. Archived version: 10.5281/zenodo.10276703 Operating system(s): platform independent but software requirements to be fulfilled Programming language: Python3.8, R for shiny-app Other requirements: biopython, pandas, joblib, SignalP4.1,TMHMM2.0, MobiDB-lite1.0, Prosite1.86, singularity3.7 (at least) License: e.g. GNU GPL LEAPH, along with training sequences, feature tables and the Shiny app are available on GitHub at: https://github.com/Plant-Net/LEAPH-EffectorComb.git. The pre-trained LEAPH and the Shiny app are also available as a definition file for container usage (Singularity 3.7 recommended). Data are available in the Zenodo repository: 10.5281/zenodo.10276703

## References

[1] Leisner C.P., Potnis N., Sanz-Saez A. Crosstalk and trade-offs: plant responses to climate change-associated abiotic and biotic stresses. Plant Cell Environ. 2023; 46:2946–2963. 36585762 10.1111/pce.14532

[2] Allwood J.W., Williams A., Uthe H., van Dam N.M., Mur L.A.J., Grant M.R., Pétriacq P. Unravelling plant responses to stress—the importance of targeted and untargeted metabolomics. Metabolites. 2021; 11:558. 34436499 10.3390/metabo11080558 PMC8398504

[3] Sato K., Kadota Y., Shirasu K. Plant immune responses to parasitic nematodes. Front. Plant Sci. 2019; 10:1165. 31616453 10.3389/fpls.2019.01165 PMC6775239

[4] Hutin M., Pérez-Quintero A.L., Lopez C., Szurek B. MorTAL Kombat: the story of defense against TAL effectors through loss-of-susceptibility. Front. Plant Sci. 2015; 6:535. 26236326 10.3389/fpls.2015.00535 PMC4500901

[5] Misas-Villamil J.C., van der Hoorn R.A.L., Doehlemann G. Papain-like cysteine proteases as hubs in plant immunity. New Phytol. 2016; 212:902–907. 27488095 10.1111/nph.14117

[6] Gorshkov V., Tsers I. Plant susceptible responses: the underestimated side of plant–pathogen interactions. Biol. Rev. 2022; 97:45–66. 34435443 10.1111/brv.12789 PMC9291929

[7] Attard A., Evangelisti E., Kebdani-Minet N., Panabières F., Deleury E., Maggio C., Ponchet M., Gourgues M. Transcriptome dynamics of *Arabidopsis thaliana* root penetration by the oomycete pathogen *Phytophthora parasitica*. BMC Genomics. 2014; 15:538. 24974100 10.1186/1471-2164-15-538 PMC4111850

[8] Shukla N., Yadav R., Kaur P., Rasmussen S., Goel S., Agarwal M., Jagannath A., Gupta R., Kumar A. Transcriptome analysis of root-knot nematode (*Meloidogyne incognita*)-infected tomato (*Solanum lycopersicum*) roots reveals complex gene expression profiles and metabolic networks of both host and nematode during susceptible and resistance responses. Mol. Plant Pathol. 2018; 19:615–633. 28220591 10.1111/mpp.12547 PMC6638136

[9] Jaouannet M., Morris J.A., Hedley P.E., Bos J.I.B. Characterization of Arabidopsis transcriptional responses to different aphid species reveals genes that contribute to host susceptibility and non-host resistance. PLoS Pathog. 2015; 11:e1004918. 25993686 10.1371/journal.ppat.1004918 PMC4439036

[10] Galán J.E. Common themes in the design and function of bacterial effectors. Cell Host Microbe. 2009; 5:571–579. 19527884 10.1016/j.chom.2009.04.008 PMC2729653

[11] Chen Y.F., Xia Y. Structural profiling of bacterial effectors reveals enrichment of host-interacting domains and motifs. Front Mol. Biosci. 2021; 8:626600. 34012977 10.3389/fmolb.2021.626600 PMC8126662

[12] Langin G., Gouguet P., Üstün S. Microbial effector proteins—a journey through the proteolytic landscape. Trends Microbiol. 2020; 28:523–535. 32544439 10.1016/j.tim.2020.02.010

[13] Stergiopoulos I., de Wit P.J.G.M. Fungal effector proteins. Annu. Rev. Phytopathol. 2009; 47:233–263. 19400631 10.1146/annurev.phyto.112408.132637

[14] Carreón-Anguiano K.G., Vila-Luna S.E., Sáenz-Carbonell L., Canto-Canché B. Novel insights into phytoplasma effectors. Horticulturae. 2023; 9:1228.

[15] Nur M., Wood K., Michelmore R. EffectorO: motif-independent prediction of effectors in oomycete genomes using machine learning and lineage specificity. Mol. Plant Microbe Interact. 2023; 36:397–410. 36853198 10.1094/MPMI-11-22-0236-TA

[16] Sperschneider J., Gardiner D.M., Dodds P.N., Tini F., Covarelli L., Singh K.B., Manners J.M., Taylor J.M. EffectorP: predicting fungal effector proteins from secretomes using machine learning. New Phytol. 2016; 210:743–761. 26680733 10.1111/nph.13794

[17] Sperschneider J., Dodds P.N., Gardiner D.M., Singh K.B., Taylor J.M. Improved prediction of fungal effector proteins from secretomes with EffectorP 2.0. Mol. Plant Pathol. 2018; 19:2094–2110. 29569316 10.1111/mpp.12682 PMC6638006

[18] Sperschneider J., Dodds P.N. EffectorP 3.0: prediction of apoplastic and cytoplasmic effectors in fungi and oomycetes. Mol. Plant Microbe Interact. 2022; 35:146–156. 34698534 10.1094/MPMI-08-21-0201-R

[19] Kristianingsih R., MacLean D. Accurate plant pathogen effector protein classification ab initio with deepredeff: an ensemble of convolutional neural networks. BMC Bioinform. 2021; 22:372. 10.1186/s12859-021-04293-3 PMC8285798 34273967

[20] Jing R., Wen T., Liao C., Xue L., Liu F., Yu L., Luo J. DeepT3 2.0: improving type III secreted effector predictions by an integrative deep learning framework. NAR Genom. Bioinform. 2021; 3:lqab086. 34617013 10.1093/nargab/lqab086 PMC8489581

[21] Arnold R., Brandmaier S., Kleine F., Tischler P., Heinz E., Behrens S., Niinikoski A., Mewes H.-W., Horn M., Rattei T. Sequence-based prediction of type III secreted proteins. PLoS Pathog. 2009; 5:e1000376. 19390696 10.1371/journal.ppat.1000376 PMC2669295

[22] Kumari S., Nagendran K., Rai A.B., Singh B., Rao G.P., Bertaccini A. Global status of phytoplasma diseases in vegetable crops. Front. Microbiol. 2019; 10:1349. 31316474 10.3389/fmicb.2019.01349 PMC6610314

[23] Lee I.-M., Davis R.E., Gundersen-Rindal D.E. Phytoplasma: phytopathogenic mollicutes. Annu. Rev. Microbiol. 2000; 54:221–255. 11018129 10.1146/annurev.micro.54.1.221

[24] Hogenhout S.A., Oshima K., Ammar E.-D., Kakizawa S., Kingdom H.N., Namba S. Phytoplasmas: bacteria that manipulate plants and insects. Mol. Plant Pathol. 2008; 9:403–423. 18705857 10.1111/j.1364-3703.2008.00472.x PMC6640453

[25] Bertaccini A., Oshima K., Kube M., Rao G.P. Phytoplasmas: Plant Pathogenic Bacteria - III: Genomics, Host Pathogen Interactions and Diagnosis. 2019; Singapore Springer.

[26] Iwabuchi N., Endo A., Kameyama N., Satoh M., Miyazaki A., Koinuma H., Kitazawa Y., Maejima K., Yamaji Y., Oshima K. et al. First report of ‘*Candidatus* Phytoplasma malaysianum’ associated with *Elaeocarpus* yellows of *Elaeocarpus zollingeri*. J. Gen. Plant Pathol. 2018; 84:160–164.

[27] Strauss E. Microbiology. Phytoplasma research begins to bloom. Science. 2009; 325:388–390. 19628836 10.1126/science.325_388

[28] Brown S.E., Been B.O., McLaughlin W.A. First report of lethal yellowing group (16Sr IV) of phytoplasmas in *Vernonia cinerea* in Jamaica. Plant Dis. 2008; 92:1132–1132. 10.1094/PDIS-92-7-1132A 30769504

[29] Bertaccini A. Phytoplasmas: diversity, taxonomy, and epidemiology. Front. Biosci. 2007; 12:673–689. 17127328 10.2741/2092

[30] Arashida R., Kakizawa S., Ishii Y., Hoshi A., Jung H.-Y., Kagiwada S., Yamaji Y., Oshima K., Namba S. Cloning and characterization of the antigenic membrane protein (Amp) gene and in situ detection of Amp from malformed flowers infected with Japanese hydrangea phyllody phytoplasma. Phytopathology. 2008; 98:769–775. 18943252 10.1094/PHYTO-98-7-0769

[31] Himeno M., Kitazawa Y., Yoshida T., Maejima K., Yamaji Y., Oshima K., Namba S. Purple top symptoms are associated with reduction of leaf cell death in phytoplasma-infected plants. Sci. Rep. 2014; 4:4111. 24531261 10.1038/srep04111 PMC3925944

[32] Oshima K., Ishii Y., Kakizawa S., Sugawara K., Neriya Y., Himeno M., Minato N., Miura C., Shiraishi T., Yamaji Y. et al. Dramatic transcriptional changes in an intracellular parasite enable host switching between plant and insect. PLoS One. 2011; 6:e23242. 21858041 10.1371/journal.pone.0023242 PMC3156718

[33] Namba S. Molecular and biological properties of phytoplasmas. Proc. Jpn Acad. Ser. B Phys. Biol. Sci. 2019; 95:401–418. 10.2183/pjab.95.028 PMC6766451 31406061

[34] Jarausch B., Tedeschi R., Sauvion N., Gross J., Jarausch W. Bertaccini A., Weintraub P.G., Rao G.P., Mori N. Psyllid vectors. Phytoplasmas: Plant Pathogenic Bacteria - II: Transmission and Management of Phytoplasma-Associated Diseases. 2019; Singapore Springer 53–78.

[35] Oshima K., Maejima K., Isobe Y., Endo A., Namba S., Yamaji Y. Molecular mechanisms of plant manipulation by secreting effectors of phytoplasmas. Physiol. Mol. Plant Pathol. 2023; 125:102009.

[36] Hoshi A., Oshima K., Kakizawa S., Ishii Y., Ozeki J., Hashimoto M., Komatsu K., Kagiwada S., Yamaji Y., Namba S. A unique virulence factor for proliferation and dwarfism in plants identified from a phytopathogenic bacterium. Proc. Natl Acad. Sci. USA. 2009; 106:6416–6421. 19329488 10.1073/pnas.0813038106 PMC2669400

[37] Bai X., Correa V.R., Toruño T.Y., Ammar E.-D., Kamoun S., Hogenhout S.A. AY-WB phytoplasma secretes a protein that targets plant cell nuclei. Mol. Plant Microbe Interact. 2009; 22:18–30. 19061399 10.1094/MPMI-22-1-0018

[38] Huang W., MacLean A.M., Sugio A., Maqbool A., Busscher M., Cho S.-T., Kamoun S., Kuo C.-H., Immink R.G.H., Hogenhout S.A. Parasitic modulation of host development by ubiquitin-independent protein degradation. Cell. 2021; 184:5201–5214. 34536345 10.1016/j.cell.2021.08.029 PMC8525514

[39] Pecher P., Moro G., Canale M.C., Capdevielle S., Singh A., MacLean A., Sugio A., Kuo C.-H., Lopes J.R.S., Hogenhout S.A. Phytoplasma SAP11 effector destabilization of TCP transcription factors differentially impact development and defence of Arabidopsis versus maize. PLoS Pathog. 2019; 15:e1008035. 31557268 10.1371/journal.ppat.1008035 PMC6802841

[40] MacLean A.M., Sugio A., Makarova O.V., Findlay K.C., Grieve V.M., Tóth R., Nicolaisen M., Hogenhout S.A. Phytoplasma effector SAP54 induces indeterminate leaf-like flower development in Arabidopsis plants. Plant Physiol. 2011; 157:831–841. 21849514 10.1104/pp.111.181586 PMC3192582

[41] Maejima K., Iwai R., Himeno M., Komatsu K., Kitazawa Y., Fujita N., Ishikawa K., Fukuoka M., Minato N., Yamaji Y. et al. Recognition of floral homeotic MADS domain transcription factors by a phytoplasmal effector, phyllogen, induces phyllody. Plant J. 2014; 78:541–554. 24597566 10.1111/tpj.12495 PMC4282529

[42] Liu T., Song T., Zhang X., Yuan H., Su L., Li W., Xu J., Liu S., Chen L., Chen T. et al. Unconventionally secreted effectors of two filamentous pathogens target plant salicylate biosynthesis. Nat. Commun. 2014; 5:4686. 25156390 10.1038/ncomms5686 PMC4348438

[43] Kang Q., Zhang D. Principle and potential applications of the non-classical protein secretory pathway in bacteria. Appl. Microbiol. Biotechnol. 2020; 104:953–965. 31853566 10.1007/s00253-019-10285-4

[44] Garcion C., Béven L., Foissac X. Comparison of current methods for signal peptide prediction in phytoplasmas. Front. Microbiol. 2021; 12:661524. 33841387 10.3389/fmicb.2021.661524 PMC8026896

[45] Dalio R.J.D., Herlihy J., Oliveira T.S., McDowell J.M., Machado M. Effector biology in focus: a primer for computational prediction and functional characterization. Mol. Plant Microbe Interact. 2018; 31:22–33. 29023190 10.1094/MPMI-07-17-0174-FI

[46] Carreón-Anguiano K.G., Vila-Luna S.E., Sáenz-Carbonell L., Canto-Canche B. PhyEffector, the first algorithm that identifies classical and non-classical effectors in phytoplasmas. Biomimetics (Basel). 2023; 8:550. 37999191 10.3390/biomimetics8070550 PMC10669590

[47] The UniProt Consortium UniProt: the Universal Protein Knowledgebase in 2023. Nucleic Acids Res. 2023; 51:D523–D531. 36408920 10.1093/nar/gkac1052 PMC9825514

[48] Kube M., Schneider B., Kuhl H., Dandekar T., Heitmann K., Migdoll A.M., Reinhardt R., Seemüller E. The linear chromosome of the plant-pathogenic mycoplasma ‘Candidatus Phytoplasma mali. BMC Genomics. 2008; 9:306. 18582369 10.1186/1471-2164-9-306 PMC2459194

[49] Urban M., Pant R., Raghunath A., Irvine A.G., Pedro H., Hammond-Kosack K.E. The Pathogen–Host Interactions database (PHI-base): additions and future developments. Nucleic Acids Res. 2015; 43:D645–D655. 25414340 10.1093/nar/gku1165 PMC4383963

[50] Urban M., Cuzick A., Seager J., Wood V., Rutherford K., Venkatesh S.Y., Sahu J., Iyer S.V., Khamari L., De Silva N. et al. PHI-base in 2022: a multi-species phenotype database for pathogen–host interactions. Nucleic Acids Res. 2022; 50:D837–D847. 34788826 10.1093/nar/gkab1037 PMC8728202

[51] Mukherjee S., Stamatis D., Li C.T., Ovchinnikova G., Bertsch J., Sundaramurthi J.C., Kandimalla M., Nicolopoulos P.A., Favognano A., Chen I.-M.A. et al. Twenty-five years of Genomes OnLine Database (GOLD): data updates and new features in v.9. Nucleic Acids Res. 2023; 51:D957–D963. 36318257 10.1093/nar/gkac974 PMC9825498

[52] Benson D.A., Cavanaugh M., Clark K., Karsch-Mizrachi I., Lipman D.J., Ostell J., Sayers E.W. GenBank. Nucleic Acids Res. 2013; 41:D36–D42. 23193287 10.1093/nar/gks1195 PMC3531190

[53] Nielsen H. Predicting secretory proteins with SignalP. Methods Mol. Biol. 2017; 1611:59–73. 28451972 10.1007/978-1-4939-7015-5_6

[54] Krogh A., Larsson B., von Heijne G., Sonnhammer E.L. Predicting transmembrane protein topology with a hidden Markov model: application to complete genomes. J. Mol. Biol. 2001; 305:567–580. 11152613 10.1006/jmbi.2000.4315

[55] Marín M., Uversky V.N., Ott T. Intrinsic disorder in pathogen effectors: protein flexibility as an evolutionary hallmark in a molecular arms race. Plant Cell. 2013; 25:3153–3157. 24038649 10.1105/tpc.113.116319 PMC3809524

[56] Necci M., Piovesan D., Dosztányi Z., Tosatto S.C.E. MobiDB-lite: fast and highly specific consensus prediction of intrinsic disorder in proteins. Bioinformatics. 2017; 33:1402–1404. 28453683 10.1093/bioinformatics/btx015

[57] Necci M., Piovesan D., Clementel D., Dosztányi Z., Tosatto S.C.E. MobiDB-lite 3.0: fast consensus annotation of intrinsic disorder flavors in proteins. Bioinformatics. 2021; 36:5533–5534. 33325498 10.1093/bioinformatics/btaa1045

[58] Sigrist C.J.A., Cerutti L., Hulo N., Gattiker A., Falquet L., Pagni M., Bairoch A., Bucher P. PROSITE: a documented database using patterns and profiles as motif descriptors. Brief. Bioinform. 2002; 3:265–274. 12230035 10.1093/bib/3.3.265

[59] Calia G., Porracciolo P., Kozlowski D., Schuler H., Cestaro A., Danchin E.G.J., Bottini S. Identification and characterization of specific motifs in effector proteins of plant parasites using MOnSTER. Commun. Biol. 2024; 7:850. 38992096 10.1038/s42003-024-06515-9 PMC11239862

[60] Breiman L. Random forests. Machine Learning. 2001; 45:5–32.

[61] Chen T., Guestrin C. XGBoost: a scalable tree boosting system. Proceedings of the 22nd ACM SIGKDD International Conference on Knowledge Discovery and Data Mining, KDD ’16. 2016; NY Association for Computing Machinery 785–794.

[62] M. Bishop C. Pattern Recognition and Machine Learning. 2006; NY Springer.

[63] Manning C.D., Raghavan P., Schütze H. Introduction to Information Retrieval. 2008; Cambridge Cambridge University Press.

[64] Pedregosa F., Varoquaux G., Gramfort A., Michel V., Thirion B., Grisel O., Blondel M., Prettenhofer P., Weiss R., Dubourg V. et al. Scikit-learn: Machine Learning in Python. JMLR. 2011; 12:2825–2830.

[65] Walsh I., Fishman D., Garcia-Gasulla D., Titma T., Pollastri G., Machine Learning Focus Group E.L.I.X.I.R., Harrow J., Psomopoulos F.E., Tosatto S.C.E DOME: recommendations for supervised machine learning validation in biology. Nat. Methods. 2021; 18:1122–1127. 34316068 10.1038/s41592-021-01205-4

[66] Käll L., Krogh A., Sonnhammer E.L.L. A combined transmembrane topology and signal peptide prediction method. J. Mol. Biol. 2004; 338:1027–1036. 15111065 10.1016/j.jmb.2004.03.016

[67] Camacho C., Coulouris G., Avagyan V., Ma N., Papadopoulos J., Bealer K., Madden T.L. BLAST+: architecture and applications. BMC Bioinformatics. 2009; 10:421. 20003500 10.1186/1471-2105-10-421 PMC2803857

[68] Bendtsen J.D., Kiemer L., Fausbøll A., Brunak S. Non-classical protein secretion in bacteria. BMC Microbiol. 2005; 5:58. 16212653 10.1186/1471-2180-5-58 PMC1266369

[69] Lundberg S.M., Lee S.-I. A unified approach to interpreting model predictions. Advances in Neural Information Processing Systems. 2017; 30:NIPS'17: Proceedings of the 31st International Conference on Neural Information Processing Systems 4768–4777.

[70] Shen W., Le S., Li Y., Hu F. SeqKit: a cross-platform and ultrafast toolkit for FASTA/Q file manipulation. PLoS One. 2016; 11:e0163962. 27706213 10.1371/journal.pone.0163962 PMC5051824

[71] Jones P., Binns D., Chang H.-Y., Fraser M., Li W., McAnulla C., McWilliam H., Maslen J., Mitchell A., Nuka G. et al. InterProScan 5: genome-scale protein function classification. Bioinformatics. 2014; 30:1236–1240. 24451626 10.1093/bioinformatics/btu031 PMC3998142

[72] Kohonen T. Self-Organizing Maps. 2001; Berlin, Heidelberg Springer.

[73] Kluyver T., Ragan-Kelley B., Pérez F., Granger B., Bussonnier M., Frederic J., Kelley K., Hamrick J., Grout J., Corlay S. et al. Loizides F., Scmidt B. Jupyter Notebooks—a publishing format for reproducible computational workflows. Positioning and Power in Academic Publishing: Players, Agents and Agendas. 2016; Amsterdam IOS Press 87–90.

[74] Kurtzer G.M., Sochat V., Bauer M.W. Singularity: scientific containers for mobility of compute. PLoS One. 2017; 12:e0177459. 28494014 10.1371/journal.pone.0177459 PMC5426675

[75] Gao X., Ren Z., Zhao W., Li W. *Candidatus* Phytoplasma ziziphi encodes non-classically secreted proteins that suppress hypersensitive cell death response in *Nicotiana benthamiana*. Phytopathol. Res. 2023; 5:11.

[76] Jomantiene R., Zhao Y., Davis R.E. Sequence-variable mosaics: composites of recurrent transposition characterizing the genomes of phylogenetically diverse phytoplasmas. DNA Cell Biol. 2007; 26:557–564. 17688407 10.1089/dna.2007.0610

[77] Wang N., Li Y., Chen W., Yang H.Z., Zhang P.H., Wu Y.F. Identification of wheat blue dwarf phytoplasma effectors targeting plant proliferation and defence responses. Plant Pathol. 2018; 67:603–609.

[78] Kanja C., Hammond-Kosack K.E. Proteinaceous effector discovery and characterization in filamentous plant pathogens. Mol. Plant Pathol. 2020; 21:1353–1376. 32767620 10.1111/mpp.12980 PMC7488470

[79] Bai B., Zhang G., Pei B., Song Q., Hao X., Zhao L., Wu Y. The function of the phytoplasma effector SWP12 depends on the properties of two key amino acids. J. Biol. Chem. 2023; 299:103052. 36813236 10.1016/j.jbc.2023.103052 PMC10040895

[80] Wang L., Chen W., Ma H., Li J., Hao X., Wu Y. Identification of RNA silencing suppressor encoded by wheat blue dwarf (WBD) phytoplasma. Plant Biol. (Stuttg.). 2021; 23:843–849. 33749977 10.1111/plb.13257

[81] Seemüller E., Sule S., Kube M., Jelkmann W., Schneider B. The AAA+ ATPases and HflB/FtsH proteases of ‘*Candidatus* Phytoplasma mali’: phylogenetic diversity, membrane topology, and relationship to strain virulence. MPMI. 2013; 26:367–376. 23387471 10.1094/MPMI-09-12-0221-R

[82] Seemüller E., Kampmann M., Kiss E., Schneider B. HflB gene-based phytopathogenic classification of ‘*Candidatus* Phytoplasma mali’ strains and evidence that strain composition determines virulence in multiply infected apple trees. Mol. Plant Microbe Interact. 2011; 24:1258–1266. 21899439 10.1094/MPMI-05-11-0126

[83] Akhtar A.A., Turner D.P.J The role of bacterial ATP-binding cassette (ABC) transporters in pathogenesis and virulence: therapeutic and vaccine potential. Microb. Pathog. 2022; 171:105734. 36007845 10.1016/j.micpath.2022.105734

[84] Zeng Y., Charkowski A.O. The role of ATP-binding cassette transporters in bacterial phytopathogenesis. Phytopathology. 2021; 111:600–610. 33225831 10.1094/PHYTO-06-20-0212-RVW

[85] Schwan W.R., Warrener P., Keunz E., K. Stover C., Folger K.R Mutations in the *cueA* gene encoding a copper homeostasis P-type ATPase reduce the pathogenicity of *Pseudomonas aeruginosa* in mice. Int. J. Med. Microbiol. 2005; 295:237–242. 16128398 10.1016/j.ijmm.2005.05.005

[86] Shi X., Festa R.A., Ioerger T.R., Butler-Wu S., Sacchettini J.C., Darwin K.H., Samanovic M.I. The copper-responsive RicR regulon contributes to *Mycobacterium tuberculosis* virulence. mBio. 2014; 5:e00876-13. 24549843 10.1128/mBio.00876-13 PMC3944814

[87] Wolschendorf F., Ackart D., Shrestha T.B., Hascall-Dove L., Nolan S., Lamichhane G., Wang Y., Bossmann S.H., Basaraba R.J., Niederweis M. Copper resistance is essential for virulence of *Mycobacterium tuberculosis*. Proc. Natl Acad. Sci. USA. 2011; 108:1621–1626. 21205886 10.1073/pnas.1009261108 PMC3029754

[88] Johnson M.D.L., Kehl-Fie T.E., Klein R., Kelly J., Burnham C., Mann B., Rosch J.W. Role of copper efflux in pneumococcal pathogenesis and resistance to macrophage-mediated immune clearance. Infect. Immun. 2015; 83:1684–1694. 25667262 10.1128/IAI.03015-14 PMC4363445

[89] Ladomersky E., Khan A., Shanbhag V., Cavet J.S., Chan J., Weisman G.A., Petris M.J. Host and pathogen copper-transporting P-type ATPases function antagonistically during *Salmonella* infection. Infect. Immun. 2017; 85:e00351-17. 28652309 10.1128/IAI.00351-17 PMC5563570

[90] Tomkins M., Kliot A., Marée A.F., Hogenhout S.A. A multi-layered mechanistic modelling approach to understand how effector genes extend beyond phytoplasma to modulate plant hosts, insect vectors and the environment. Curr. Opin. Plant Biol. 2018; 44:39–48. 29547737 10.1016/j.pbi.2018.02.002

[91] Lu Q., Li S., Shao F. Sweet talk: protein glycosylation in bacterial interaction with the host. Trends Microbiol. 2015; 23:630–641. 26433695 10.1016/j.tim.2015.07.003

[92] Arricau-Bouvery N., Duret S., Dubrana M.-P., Desqué D., Eveillard S., Brocard L., Malembic-Maher S., Foissac X. Interactions between the flavescence dorée phytoplasma and its insect vector indicate lectin-type adhesion mediated by the adhesin VmpA. Sci. Rep. 2021; 11:11222. 34045641 10.1038/s41598-021-90809-z PMC8160148

[93] Nutricati E., De Pascali M., Negro C., Bianco P.A., Quaglino F., Passera A., Pierro R., Marcone C., Panattoni A., Sabella E. et al. Signaling cross-talk between salicylic and gentisic acid in the ‘*Candidatus* Phytoplasma solani’ interaction with Sangiovese vines. Plants. 2023; 12:2695. 37514309 10.3390/plants12142695 PMC10383235

[94] Bertazzon N., Bagnaresi P., Forte V., Mazzucotelli E., Filippin L., Guerra D., Zechini A., Cattivelli L., Angelini E. Grapevine comparative early transcriptomic profiling suggests that Flavescence dorée phytoplasma represses plant responses induced by vector feeding in susceptible varieties. BMC Genomics. 2019; 20:526. 31242866 10.1186/s12864-019-5908-6 PMC6595628

[95] Meinnel T., Giglione C. Tools for analyzing and predicting N-terminal protein modifications. Proteomics. 2008; 8:626–649. 18203265 10.1002/pmic.200700592

[96] Meinnel T., Giglione C. Protein lipidation meets proteomics. Front. Biosci. 2008; 13:6326–6340. 18508663 10.2741/3157

[97] Zha J., Weiler S., Oh K.J., Wei M.C., Korsmeyer S.J. Posttranslational N-myristoylation of BID as a molecular switch for targeting mitochondria and apoptosis. Science. 2000; 290:1761–1765. 11099414 10.1126/science.290.5497.1761

[98] Medina-Puche L., Tan H., Dogra V., Wu M., Rosas-Diaz T., Wang L., Ding X., Zhang D., Fu X., Kim C. et al. A defense pathway linking plasma membrane and chloroplasts and co-opted by pathogens. Cell. 2020; 182:1109–1124. 32841601 10.1016/j.cell.2020.07.020

[99] Wang N., Yang H., Yin Z., Liu W., Sun L., Wu Y. Phytoplasma effector SWP1 induces witches’ broom symptom by destabilizing the TCP transcription factor BRANCHED1. Mol. Plant Pathol. 2018; 19:2623–2634. 30047227 10.1111/mpp.12733 PMC6638060

[100] Kitazawa Y., Iwabuchi N., Himeno M., Sasano M., Koinuma H., Nijo T., Tomomitsu T., Yoshida T., Okano Y., Yoshikawa N. et al. Phytoplasma-conserved phyllogen proteins induce phyllody across the Plantae by degrading floral MADS domain proteins. J. Exp. Bot. 2017; 68:2799–2811. 28505304 10.1093/jxb/erx158 PMC5853863

[101] D’Souza S.E., Ginsberg M.H., Plow E.F Arginyl-glycyl-aspartic acid (RGD): a cell adhesion motif. Trends Biochem. Sci. 1991; 16:246–250. 1926332 10.1016/0968-0004(91)90096-e

